# Applications of Carbon Dots and Graphene Quantum Dots in Treatment of Diabetes

**DOI:** 10.3390/molecules31060941

**Published:** 2026-03-11

**Authors:** Sho Nakayama, Eric J. Shepard, Abhinandan Banerjee, Xiaoda Yang, Debbie C. Crans

**Affiliations:** 1 Department of Chemistry, Colorado State University, Fort Collins, CO 80523, USA; sho.nakayama@colostate.edu (S.N.); eric.shepard@colostate.edu (E.J.S.); abhinandan.banerjee@colostate.edu (A.B.); 2 State Key Laboratories of Natural and Mimetic Drugs and Department of Chemical Biology, School of Pharmaceutical Sciences, Peking University, Beijing 100191, China; yangxiaodapku@163.com; 3 Cell and Molecular Biology Program, Colorado State University, Fort Collins, CO 80523, USA

**Keywords:** Diabetes, carbon nanoparticles, carbon dots, graphene quantum dots, carbon quantum dots, animal studies, in vitro, in vivo, drug delivery system, insulin, metformin, glimepiride, vanadium antidiabetic agents, blood glucose

## Abstract

Carbon nanoparticles (CNPs) are increasingly being considered for medical applications. The objective of this article is to determine which anti-diabetic drugs and compounds have been enhanced by CNPs, and which CNP scaffolds were found to be successful. The anti-diabetic drugs administered loaded on CNPs include insulin, metformin, glimepiride and vanadium compounds. Carbon quantum dots (CQDs), graphene quantum dots (GQDs), graphene oxide quantum dots (GOQDs), hybrid systems and fullerenes are all carriers able to alleviate symptoms of diabetes. Successful CNPs are 10 nm or less and can have a flat pancake structure, as well as the spherical CQDs and the spherical-but-hollow gadofullerene (Gd-C82). The use of the carbon nanoparticle scaffold includes oral, intravenous administration and placement as an implant in a diabetic animal model system. In vitro studies in an insulin-resistant model demonstrate a 500–1000-fold enhancement of metformin when placed on the pegylated GOQD. Although some CNPs have low toxicity, more information is needed for understanding the metabolism associated with uptake and processing. In summary, CNPs represent a novel class of nanoparticles that has promising potential. They enhance the efficacy of anti-diabetic drugs, have low toxicity, and keep the loaded drug protected until reaching their targets.

## 1. Introduction

Diabetes mellitus (DM) is a chronic metabolic disorder characterized by elevated blood glucose levels resulting from defects in insulin production and/or from acquired insulin resistance [1]. The number of patients with DM continues to rise globally, and in 2024, approximately 589 million adults aged 20 to 79 years were living with DM [2]. By 2050, the number is projected to increase by 45%, to 853 million worldwide [2]. Currently, the known therapeutic regime for the treatment of DM includes insulin, metformin, GLP receptor agonists, DPP4 inhibitors, thiazolidinediones, meglitinides, SGLT2 inhibitors, α-glucosidase inhibitors, with the choice of medication being dependent on Type 1 or Type 2 diabetes mellitus [3,4]. The search for better drugs for the treatment of DM continues, as well as improved and novel administration methods for drugs in general [5,6,7,8,9,10]. Type 1 diabetes mellitus is an autoimmune condition characterized by insulin deficiency, typically requiring lifelong insulin therapy and often occurs in childhood or adolescence, although onset in adulthood is possible [11,12]. Type 2 diabetes mellitus, by contrast, is driven primarily by insulin resistance often accompanied by progressive impairment of β-cell function and its risk factors include age, obesity, and lack of physical activity [12]. Type 2 diabetes mellitus accounts for about 90–95% of all diabetes cases globally, while Type 1 diabetes mellitus makes up the remaining 5–10% [13]. The rising prevalence of diabetes, particularly Type 2 diabetes mellitus, poses significant challenges for public health and healthcare systems worldwide, especially given the associated risks of complications such as cardiovascular disease, kidney failure, blindness and amputation [12]. In this work, we investigate the reported effects of carbon-based nanocarriers for anti-diabetic drugs as part of delivery platforms.

Nanostructures typically have 2–3 dimensions within the 1–100 nm range, and, at this small scale, they have unique properties which can be desirable when used for biological applications. Specifically, their physicochemical, distribution, composition and material properties will differ from corresponding structures that exist in bulk. In this review we shall focus exclusively on zero-dimensional nanostructures, that is, structures that are nanosized in all three dimensions, also referred to as nanoparticles (NPs). Many NPs exist, including rigid and shape-preserving nanoparticles, “hard NPs” that differ from deformable and hydrated assemblies, and “soft NPs”. Considering the varied types of NPs, it is not surprising that methods for preparation of NPs also vary dramatically [14,15]. In recent years, advances in NP synthesis have enabled us to prepare NP systems of particular sizes, composition and surface properties. Each nanoparticle system has its advantages, whether it be convenience or expense of preparation, solubility, ability to carry a cargo, or tunable toxicity to cellular systems. Diabetes is a life-long chronic disease, requiring anti-diabetic medications (including their carriers) to be efficacious, cost-effective, and non-toxic both in the short term and upon prolonged exposure [16,17]. Carbon-based nanoparticles (CNPs) are a group of nanoparticles that are synthesized or prepared by carbon sources such as citric acid, glucose, carbon nanotubes, and graphite [18,19,20], and their applications are less explored compared to other types of nanoparticles [21,22,23]. However, we have reported successful applications of CNPs with some anti-diabetic vanadium compounds and we were interested in how much CNPs have been used for treatment of diabetes [6,7].

**Figure 1:**
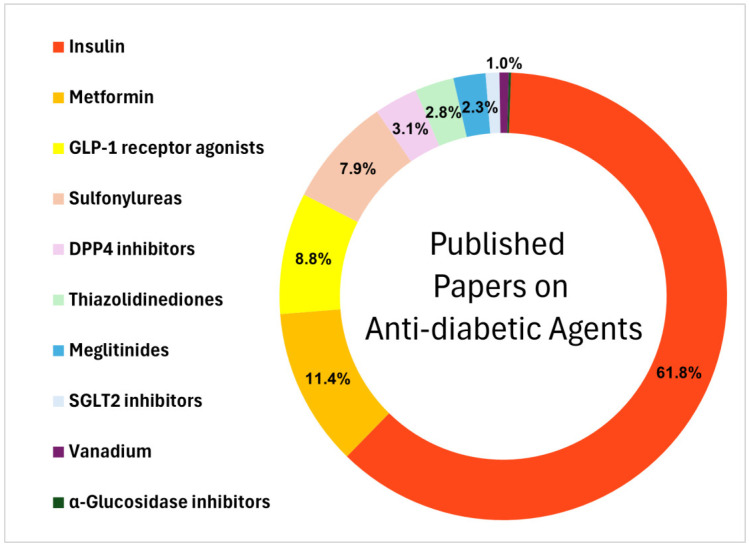
A pie chart showing the proportion of anti-diabetic agents (insulin, metformin, GLP-1 receptor agonists, sulfonylureas, DPP4 inhibitors, thiazolidinediones, meglitinides, SGLT2 inhibitors, vanadium, and a few minor drugs such as α-glucosidase inhibitors to bring the total to 100%) delivered on nanoparticle carriers as found using the databases Web of Science, SciFinder, Google Scholar, and Scopus (no limitation on time-period).

Conventional drug delivery systems, both oral and injectable forms, have therapeutic limitations including uncontrolled release, poor bioavailability, and unknown long-term toxicity [8,20]. Nanoparticle-mediated drug delivery has emerged as a promising approach, providing cutting-edge strategies to improve a drug’s solubility, stability, selectivity, and release profile [24,25,26,27,28]. For instance, the first FDA-approved nanodrug, doxorubicin hydrochloride liposome, was developed in 1995 and showed improved site-selectivity and reduced toxicity compared to doxorubicin hydrochloride [24,29]. Since then, the field of nanomedicine has been growing rapidly, offering innovative solutions to health conditions such as diabetes, cancers, cardiovascular disorders, infectious diseases, and neurodegenerative diseases [30,31,32]. In Figure 1, we show classes of anti-diabetic agents that have been delivered using any type of nanoparticle including metal-based nanoparticles, carbon-based nanoparticles, liposomal-based nanoparticles and polymeric nanoparticles. According to our literature research, insulin occupied the majority of NP-mediated drug-loading papers (61.8%), followed by metformin (11.4%), GLP-1 receptor agonist (8.8%) and sulfonylureas (7.9%), which altogether represent about 90% of the published papers. On the other end of the scale, preclinical studies with vanadium represent only 1% of the cases reported. The goal of this review is to investigate the progresses made by anti-diabetic drugs loaded on different CNP-mediated drug delivery systems and compare their respective advantages and disadvantages seen from studies to assist future development in this field.

## 2. Known Anti-Diabetic Agents Loaded on Carbon Nanoparticles

**Figure 2:**
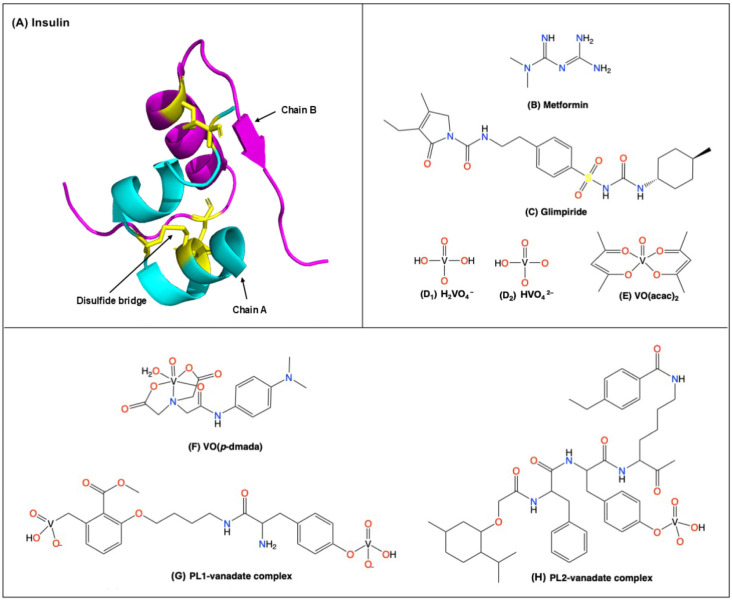
Insulin (**A**); metformin (**B**); glimepiride (**C**); vanadate: H_2_VO_4_^−^ (**D_1_**) and HVO_4_^2−^ (**D_2_**), VO (acac)_2_ (**E**); VO(*p*-dmada) (**F**); vanadate + PL1 (**G**); and vanadate + PL2 (**H**).

Carbon nanoparticles as a class of carriers have been less explored than other types of nanoparticles such as liposomes, metal-based nanoparticles, and polymeric nanoparticles. Selected anti-diabetic agents that have been reported for their drug-loading with CNPs are summarized in this section and listed in Figure 2.

Sufficient insulin (Figure 2A) levels are necessary for maintaining euglycemia because insulin signaling is critical for maintaining healthy glucose levels and facilitating glucose uptake and metabolism [33]. For Type 1 diabetes mellitus, insufficient insulin production requires frequent administration of insulin. Type 2 diabetes mellitus is associated with dysfunctions in both production and recognition of insulin, and administration of insulin can be effective for some patients [34]. However, insulin is a protein, and like many naturally occurring protein therapeutics, it is rapidly metabolized and cleared in vivo, which limits its biological half-life and poses challenges for sustained delivery [35]. Furthermore, insulin administration is not the first-line treatment for the majority of Type 2 diabetes mellitus patients due to the high risk of hypoglycemia and the price for chronic treatment compared to chronic treatment with metformin [36]. Conventional insulin treatment is primarily administered through subcutaneous injections, thus bypassing the stomach and avoiding digestion. Since the 1920s, attempts to develop orally administered insulin have been explored to obtain a more convenient way to administer insulin [37], and recently studies have been reported on oral insulin formulations mediated by various nanoparticles.

Metformin (Figure 2B) is the most commonly used anti-diabetic drug worldwide and is often considered a first-line medication for the treatment of Type 2 diabetes mellitus [36]. More than 200 million people are currently approximated to be using metformin [38], which is around one third of the 589 million people worldwide that live with diabetes [13]. Metformin was first approved in Europe in 1958 [39] and then in the US in 1994 [36]. Metformin is a biguanide and the only member of this class of compounds currently in use because the other biguanide derivatives, phenformin and buformin, were withdrawn in the late 1970s due to the high occurrence of lactic acidosis [40]. The structure of metformin contains several amine groups and it has a charge of +1 at neutral pH in blood and cells [41]. The calculated logarithm of the partition coefficient (CLogP) of metformin is −1.45, indicating high hydrophilicity and poor membrane permeability [42]. Although metformin already has great efficacy on its own, nanoparticle (NP)-mediated drug delivery has led to more effective formulations nowadays [43,44,45,46].

Sulfonylureas contain sulfonyl and urea groups and are insulin secretagogues. They promote insulin secretion from pancreatic β-cells, which leads to a reduction in blood glucose levels [47]. The first generation of sulfonylureas includes tolbutamide and chlorpropamide which are rarely used today due to their effects and short-acting potency [48]. The second- and third-generation sulfonylureas have a longer-acting potency and reduced side effect profiles and include gliclazide, glipizide, glibenclamide (glyburide), gliquidone, and glimepiride (Figure 2C) [48,49]. Specifically, we will focus on third-generation glimepiride which is a sulfonylurea currently in the clinic that is formulated in tablet and capsule forms [50]. However, glimepiride has a short half-life which can result in inconsistent blood levels and successful treatment which requires prolonged dosing [51]. Unfortunately, glimepiride levels that are too high can cause adverse effects such as hypoglycemia, gastrointestinal distress and visual impairments. It is a neutral molecule at pH 7.4 in blood and has a CLogP of 3.96 with poor water solubility [52]. A study using a graphene quantum dot (GQD)-mediated formulation of glimepiride reported improved efficacy and longer lifetime, maintaining blood glucose levels within a stable range for an extended period [53].

Animal studies with vanadium salt were first reported in 1899 and have continued because of the special properties of this element [54]. Vanadium is a first-row transition metal and in oxidation state five it can act as a phosphorus analog; that is, vanadate in the form of H_2_VO_4_^−^ (Figure 2D_1_) or HVO_4_^2−^ (Figure 2D_2_) is a structural and electronic analog of phosphorus H_2_PO_4_^−^ or HPO_4_^2−^ [55,56,57,58,59]. Furthermore, vanadate forms labile vanadate esters in aqueous solution [55,56,57,58]. Therefore, vanadate binds to the active sites in protein phosphatases mimicking the five-coordinate transition state of phosphate ester hydrolysis [59,60,61,62]. The evidence for the inhibition of protein tyrosine phosphatase 1B (PTP1B) and regulation of the insulin-signaling cascade [62,63] have been demonstrated by the fact that cells where the PTP1B was removed did not show any effects by vanadate [62,64]. Therefore, vanadium salts such as sodium metavanadate (NaVO_3_), sodium orthovanadate (Na_3_VO_4_), vanadyl sulfate (VOSO_4_), and coordination complexes such as bis(ethylato)oxovanadium(IV) (BEOV) [65], vanadyl acetylacetonate (VO(acac)_2_, Figure 2E), dipicolinate dioxovanadium(V) [66] and several polyoxovanadates [67] are well known to exhibit anti-diabetic activity [55]. Yang and coworkers showed effective anti-diabetic effects of VO(*p*-dmada) (Figure 2F) and two other vanadate esters (PL-1 and PL-2, Figure 2G,H) [6,7,68]. By using GQDs, hypoglycemic effects of these compounds were improved while reducing toxicity in general [7,69,70,71].

## 3. Carbon Nanoparticles Classification

CNPs in the literature have many different names depending on the shape, crystallinity, and surface chemistry including carbon nanotubes, carbon dots (CDs), carbon nanofibers, carbon nanohorns, carbon nano-onions, carbon nano diamond, fullerenes and others [19,72,73]. Overall, CNPs are typically described within a hierarchical nomenclature that combines dimensionality, core structure, and surface chemistry [74]. Unfortunately, terminology remains only partially standardized and is often inconsistent across the literature. CNPs, sometimes also referred to as CDs, are classified into subtypes such as carbon quantum dots (CQDs), also referred to as carbon nanodots (CNDs); graphene quantum dots (GQDs); carbonized polymeric dots (CPDs); and finally the class of fullerenes [75,76,77]. However, current classification schemes are often not used consistently across literature, leading to confusion in the carbon-based NP research field. In the broadest sense, “quantum dots” denotes nanoparticles with at least *one* dimension below about 10 nm [75,76,77], such that electronic states are discretized by quantum confinement which, among other phenomena, define their fluorescent properties [19]. Differences are found in particle size and crystallinity, sp^2^/sp^3^ hybridization, and the presence of graphitic layering or polymeric character [78]. CQDs are generally defined as nearly spherical, predominantly sp^2^-rich nanocrystals with a graphitic or crystalline carbon core exhibiting clear quantum-confinement-dominated photoluminescence [78]. In contrast, GQDs are anisotropic 2D flat pancake-like particles often referred to as graphene flakes, derived from graphene or related graphitic precursors, consisting of one to a few graphene layers with lateral dimensions typically below ~10 nm [19,74]. The C-atoms in graphenes are sp^2^-hybridized, but the graphene systems prepared from a bottom-up synthetic method (vide infra, §4) are not idealized; perfect graphene sheets and their bonding are best described as polyaromatic systems because of the defects in the structures including edge oxidation [79]. CDs often refer to highly carbonized, luminescent nanoparticles that lack well-resolved lattice fringes and may not show clear size-dependent quantum confinement effect [80,81]. CPDs and closely related “carbonized polymer dots” retain polymeric segments in or around the carbonaceous core, complicating strict quantum-dot classification [76,77]. Finally, the class of fullerenes essentially deviates from all the systems described above in that they are carbon allotropes with defined molecular structures forming hollow shapes such as spheres, ellipsoids, or tubes, with nanoscale dimensions in their 3D entities [82,83,84].

The proliferation of structurally overlapping categories has led to what several authors term a “nomenclature crisis”; particularly for small, amorphous, or partially graphitized particles, the terms CDs, CQDs, GQDs and graphene oxide quantum dots (GOQDs) are used interchangeably. In this manuscript we used the nomenclature guided by the crystallinity, shape, surface functionalization, emission mechanism and fluorescence [75,76,77]. To avoid confusion and address the potential nomenclature ambiguity, we subdivide the CDs into carbon quantum dots (CQDs), GQDs, and polymeric dots (CPDs). Furthermore, GQDs are subdivided into GQDs, GOQDs, and reduced graphene oxide quantum dots (rGOQD) with a variety of heteroatomic-surface functionalization available depending on the synthetic methods.

GQDs, GOQDs, and rGOQDs are a single or few layers of crystalline or amorphous materials mainly composed of sp^2^ and/or sp^3^ carbons. The structure is two-dimensional and flat, pancake-like. GQDs contain no or a minimal number of oxidized carbons corresponding to no or a few defects. The size of GQDs varies somewhat because many papers report that the particle size is either less than 10 nm [85,86] or 20 nm [87,88,89], whereas GQDs, GOQDs, and rGOQDs all fall within the same or similar particle size range [85,86,87,88,89]. The GOQD is an oxidized nanomaterial with a higher degree of oxidation, containing functional groups such as carboxylic acids, epoxides, and alcohols. Unfortunately, some publications applying these materials as drug delivery vehicles do not report the data (size and preparation methods) for these systems, not allowing for the determination of which sub-group of the carbon nanoparticle the scaffold belong to. In this review, we have defined CNPs and their subtypes as the following: CNPs as the largest umbrella to encompass all types of carbon-based nanoparticles, CDs as a subtype of CNPs, and CQDs/GQDs as further subcategories of CDs. We have also classified/reclassified the specific CD types using the method of preparation described by authors of the publications; different preparations generally lead to different, predictable types of CDs. Specifically, a bottom-up synthesis of the NP, unless data is provided to show otherwise, may be better described as a CQD instead of a GQD or GOQD, even if the authors have suggested the latter.

## 4. Carbon Nanoparticle Synthesis

**Figure 3:**
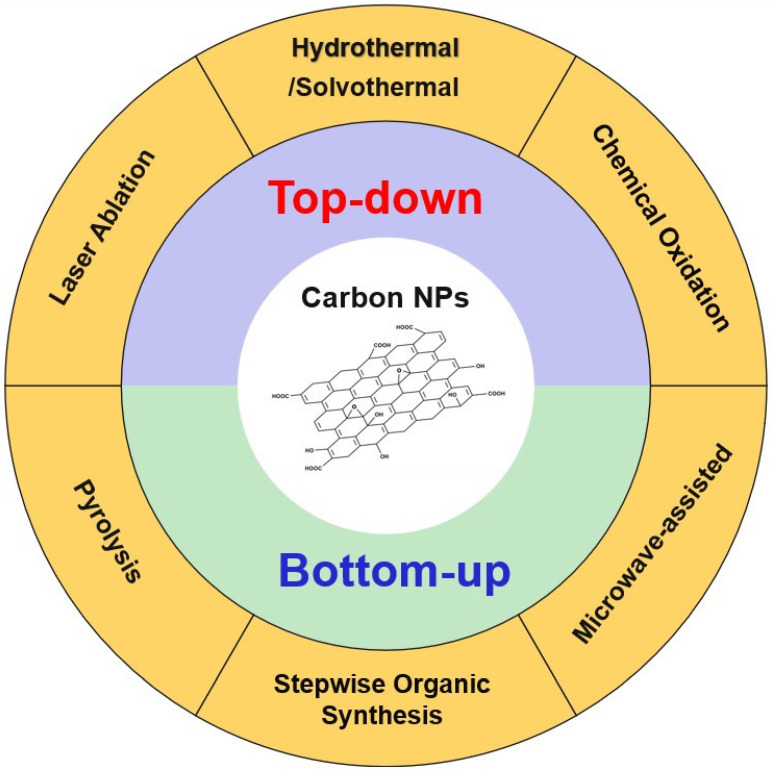
Top-down and bottom-up methods with different techniques for the synthesis of CNPs. A small image shown representing CNP and described in detail in Section 5.

In the preparation of GQDs, there are two fundamentally different methods: top-down and bottom-up methods illustrated in Figure 3. Top-down methods break carbon-rich bulk materials such as graphite, carbon nanotubes, and fullerene [90,91] down to nano-sized particles. The methods used for the preparation depend on what they are going to be used for and the desired size of the NP. Although there are exceptions, the top-down method often involves making large NPs, and one can generalize the sizes to be as follows: ball milling methods generally result in NPs from about 20–200 nm; arc discharge methods generally form NPs about 10–100 nm [92]; ultrasonication methods form NPs about 5–50 nm [93]; and exfoliation methods generally form NPs about 5–100 nm [93]. In contrast to the other top-down methods, laser ablation generally forms smaller NPs ranging from about 2–20 nm [94]. Fullerene syntheses have developed dramatically since their discovery but can now be prepared readily from vapor deposition methods. Furthermore, many approaches have been developed, and modified systems are now available [82,83,84]. Considering the main scope of this review is not the synthesis of CNPs, we have kept this section brief and refer readers to the related references for more information.

Bottom-up methods prepare nanoparticles by assembling molecular precursors such as citric acid or glucose in the presence of potential dopant molecules [90,91,95]. These methods involve condensation/polymerization reactions. The nucleation of the particles can be facilitated by chemical vapor deposition, pyrolysis, hydrothermal synthesis, microwave-assisted synthesis and flame synthesis/combustion. The sizes typically formed by these methods can be in the ranges of μm to nm depending on the synthetic method used. In general, bottom-up methods offer better compositional control and surface tunability than top-down methods. However, bottom-up methods prepare particles that contain both sp^2^ and sp^3^ carbon atoms, hence the sp^2^ regions of the particles can serve as a platform for drugs within the overall CD structure. Heteroatom doping, using nitrogen, oxygen, sulfur, and/or phosphorus precursors to modify the CNDs, can increase their stability and water solubility [96,97,98]. This is very important for the stability of the particles, because the particles can continue to assemble and increase in size in solution over time. Heteroatom doping increases the zeta potential (surface charges), leading to particle repulsions and thus avoiding continuous aggregation of the NPs and stabilizing their sizes. Heteroatom doping can also increase photoluminescence and electrical conductivity of CNDs, providing desirable properties for theranostic applications and various types of biological treatments using photothermal methods. Heteroatom doping can also improve the ability of the NP to associate with a drug and can be a key parameter that determines the loading of a drug and consequently its potency.

Since preparing pristine GQDs on a large scale is not trivial and preparation often results in oxidized graphene (GOQD), use of this material is direct and economically more attractive [99,100]. As a result, preparation of pure GQDs with a lower degree of oxidation is now often performed using reducing agents and is referred to as rGOQD. Reducing GOQDs is a much easier and more affordable method to prepare GQDs than direct methods. Furthermore, rGOQDs have similar structural characteristics to pristine GQDs, with few defects or holes [101,102].

## 5. Drug Loading of Carbon Nanoparticles: General Comments

**Figure 4:**
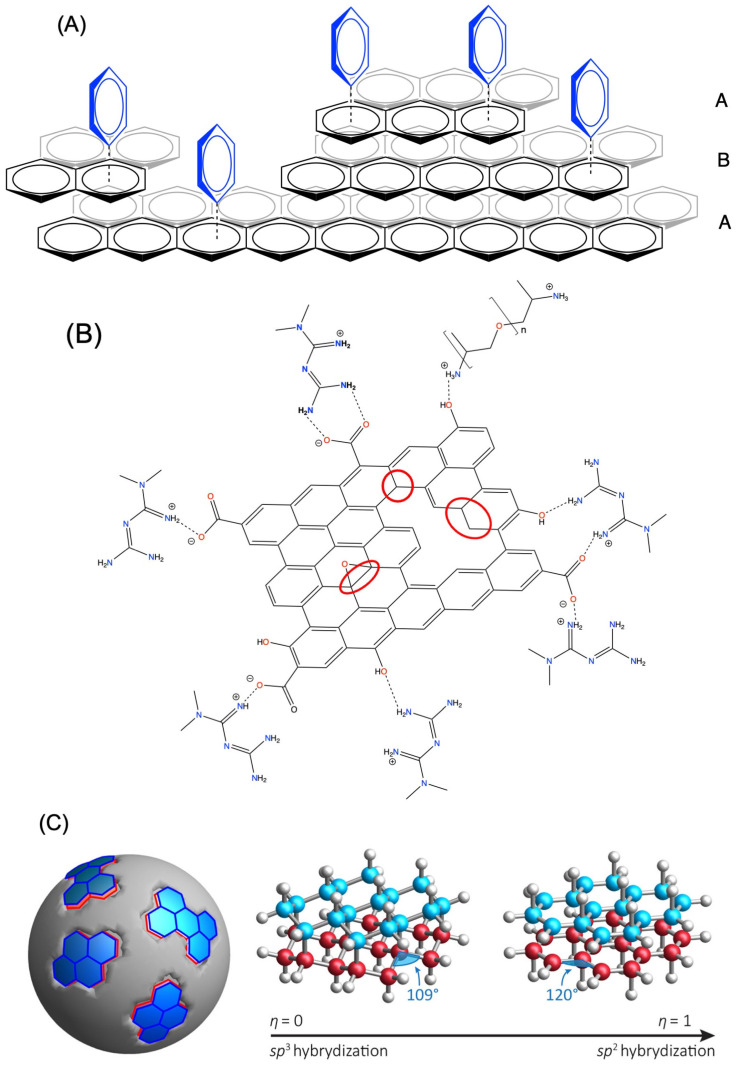
The black and gray edge plane (three layers labeled A, B, A) of non-pristine graphene is shown. The blue benzene molecules represent drug molecules. The π-π stacking between electron-rich drug and the electron-rich basal plane of non-pristine graphene is indicated by the dotted line (**A**). The basal plane of a pegylated GOQD is shown, containing carbon vacancies, oxygen functional groups (such as carboxylates, alcohols, epoxides, and carbonyls), and sections of unsaturated sp^3^ carbons (circled in red). The most prominent form of protonated metformin is shown loaded on a GOQD-PEG system via H-bond interactions and salt bridges (**B**). The image of a spherical CQD containing both sp^3^ and sp^2^ carbon atoms on its surface is shown to the left in (**C**). In the center figure of (**C**) the structure of a sp^3^ domain with diamond-like structure of joined six-membered rings in chair conformations is shown; hydrogen atoms are shown as white spheres, top layer of carbon atoms are shown in blue, and bottom layer of carbon atoms are shown in red. To the right of figure (**C**), the structure of a sp^2^ domain with graphite structure is shown; hydrogen atoms are white spheres, top layer of carbon atoms are in blue and bottom layer of carbon atoms are in red. Figure (**B**) published by the Public Library of Science (PLOS) in 2024 and the image is licensed under CC-BY 4.0 and a modified version is redrawn from Ref. [103]. Figure (**C**) is published by the American Chemical Society licensed under CC-BY 4.0 in 2019 from Ref. [111].

Drug loading onto carbon-based nanoparticle carriers exploits a combination of π–π stacking, hydrophobic interactions, hydrogen bonding, electrostatic complexation, and covalent conjugation to immobilize small-molecule therapeutics within or onto graphitic sp^2^-rich frameworks. CNDs including GOQD and rGOQD are largely composed of sp^2^-conjugated carbons allowing π-π stacking with the available layer in the nanoflake; schematic drug loading on the available “graphite like surface” is illustrated in Figure 4A. Experimental evidence suggests that drugs are normally loaded only on one side of the GQD sheet that is accessible, so the loading capacity on GQDs is not as high as the loading capacity of nano capsules or other encapsulated carriers such as liposomes. Nonetheless, up to 50% of the loading capacity can often be reached [103]. In Figure 4A, we show a real defective type of GQD. Even though the electronic properties of pristine GQDs are governed a band gap, experimental methods have shown that real GQDs prepared from bottom-up synthesis are generally imperfect and better described as a polyaromatic compound with a HOMO and LUMO [104,105,106]. Any interaction between a drug and the real GQD can therefore be described using molecular orbitals. The π–π interaction between the drug and the GQD is shown in Figure 4A as a benzene (mimicking drug) and the HOMO (negative π-cloud) of the GQD. As shown in Figure 4A, it is the electron-deficient side of the benzene molecule that interacts with the electron-rich π-cloud of the GQD, Figure 4A. This type of π-stacking arrangement exists between two electron-rich aromatic units [107]. If the drug molecule contained an electron-poor aromatic group, the structure would be more similar to the shifted parallel sandwich-like arrangements [107].

Heteroatom-doped CD carriers containing polar and ionizable groups such as carboxylate, amines, and sulfonate groups introduced by oxidative functionalization or additives can serve as excellent scaffolds upon which a variety of drug molecules can be loaded. These features allow for targeting drugs by modification of the GQD with additional functionalities. In the case of CDs, similar scaffolding possibilities exist, although the sp^2^ unsaturated carbon regions are less organized, containing hydrophobic saturated regions in the nanoparticle. In addition, functionalized regions can form, and those can be pH-sensitive and can be used to fine tune drug loading and drug release kinetics with pH changes under physiological or pathological conditions. In general, drug-carrier interactions for CNPs afford a binding affinity of 1000–10,000, which would give a favorable release profile with *t*_1/2_ of several hours [103].

For anti-diabetic drugs, these principles are leveraged to engineer glucose- or insulin-responsive carbon nanocarriers capable of maintaining tight glycemic control by coupling high-capacity loading with stimuli-adaptive release profiles [108]. Hydrophilic small-molecule anti-hyperglycemics (e.g., metformin, certain sulfonylureas, DPP-4 inhibitors) are frequently immobilized via ionic pairing and hydrogen bonding to oxidized or polymer-grafted carbon surfaces. More hydrophobic agents (e.g., thiazolidinediones, SGLT2 inhibitors) benefit from π–π and hydrophobic adsorption within graphitic domains or carbon nanotube interiors, which can prolong circulation and enhance oral or transdermal bioavailability [109,110].

In Figure 4B, we show an example of a GOQD scaffold modified both with polyethylene glycol (PEG) and metformin [103]. Metformin as a neutral base has a formula of C_4_H_11_N_5_, and since it is protonated at neutral pH, its cationic formula is C_4_H_12_N_5_^+^ with 6 protons on the N-atoms and 6 protons on the two methyl groups. The terminal imine group is the main N-atom that is protonated, and hence the N-atom that will form the strongest H-bond. However, this interaction can also be characterized as a salt bridge since the carboxylate is deprotonated and the imine N-atom is protonated. In the schematic image shown in Figure 4B, we show the interaction between the protonated imine group and the carboxylate. However, it should be mentioned that the two methyl groups on metformin could potentially sterically interfere thereby reducing the strength of this interaction. This is very possible because resonance stabilization makes the other end of the protonated metformin molecule also able to H-bond in a crowded area on the nanoparticle.

Incorporation of proteins such as glucose oxidase, phenylboronic-acid-containing polymers, or pH-sensitive shells onto CDs or GQDs enables “closed-loop” formulations where local hyperglycemia triggers gluconic-acid-mediated pH shifts or boronated–diol exchange, weakening carrier–drug interactions and accelerating anti-diabetic drug release in a self-regulated manner by the needs of the system [112,113]. In addition, the intrinsic photoluminescence of some CNPs allows real-time theranostic monitoring of carrier distribution and degradation, facilitating the correlation of fluorescence signals with drug release, biodistribution, and glycemic outcomes in preclinical anti-diabetic therapy models [114,115]. CNP-mediated drug loading can also be performed via covalent bonding fashion. For example, L- or D-cysteine can be covalently bonded to the carboxyl-contained CDs via amide coupling, which provides in vivo benefits in drug transport by inducing chirality and redox-sensitive linkers such as disulfide bonds [116,117,118].

## 6. Carbon Dots and Graphene Quantum Dots Loaded with SpecificAnti-Diabetic Agents

In Table 1 the following entries with key to the abbreviations and details are summarized:

Entry 1—Insulin on Hydroxylated GQD which is better described as GOQD [119]
Entry 2—The behavior of a model peptide is investigated on GOQD [120]
Entry 3—Insulin on N-doped CQD [121]
Entry 4—Metformin on Polyethylene glycol-modified GOQD (COQD-PEG), this entry is also illustrated in Figure 4B [103]
Entry 5—Metformin on hyaluronic acid-conjugated graphene oxide (GOQD-HA) [46]
Entry 6—Metformin on propylene-based hydrogel on COD [122]
Entry 7—Metformin on reduced GOD hydrogel [123]
Entry 8—Metformin on N-doped CQD hydrogel [124]
Entry 9—Glimepiride on hydroxylbutyl-Chitosan-GOD (HBC–GOQD) [53]
Entry 10—Fullerenes loaded with metformin and other drugs [5,125,126,127]
Entry 11—Fullerene C82 added Gd [125,126]
Entry 12—[VO(*p*-dmada)] on GQD [68]
Entry 13—Vanadate-PLI complex on GQD, Vanadate-PL2 complex on GQD [6]
Entry 14—Vanadyl acetylacetonate complex and 17β-oestradiol on GQD [7]

### 6.1. Insulin and Protein-Loaded Graphene Quantum Dots

Since insulin is a peptide hormone, it is subjected to cellular metabolism upon entry into cells [128]. However, the CD/GQD scaffolds have potential to protect insulin against any conformational changes or denaturation [119]. Many other NPs have been reported to be successful in protecting insulin against metabolism [8,129,130,131]. Studies with insulin on CD/GQDs are likely to be forthcoming in the near future, but at this time, we highlight the reports here with insulin on GQDs, GOQDs and N-doped CQD scaffolds (entries 1–3, in Table 1).

Human islet amyloid polypeptide (IAPP), co-secreted with insulin by pancreatic β-cells, plays dual roles in both glycemic control and the pathology of Type 2 diabetes, entry 1 in Table 1. A study was carried out with GQDs co-loaded with insulin and IAPP in pancreatic β-cells to investigate β-cell toxicity [119]. The binding between the proteins and hydroxylated GQDs was attributed to H-bonding and hydrophobic interactions as suggested from molecular dynamic simulations. GQDs without the protein cargo did not cause experimentally significant changes on the protein expression of β-cells; however, the cargo protected the damage on the pancreatic β-cells relevant to Type 2 diabetes mellitus, demonstrating that GQDs were able to protect the proteins from changing or denaturing [119].

Since drug release is an important property for a drug carrier system, the potential of using the combined hydrogel-GOQD system to obtain additional control of insulin (mass) diffusion and release were investigated [120,128]. The density of the hydrogel and the quantity of the GOQD loaded in the gel are critical parameters for modulating insulin release to better understand the diffusion of a peptide drug through the network of the GOQD membrane and GOQD-embedded hydrogels. Both theoretical and experimental approaches were used to understand and map the peptide release from the GOQD framework, showing a tunable nonlinear dependence of the peptide concentration upon time. The studies yield a model describing drug diffusion through GOQD membranes, which is extended to the diffusion of the peptide in GOQD-embedded agarose hydrogels [120]. The model demonstrates that both GOQD density and size influence the drug release rate. The ability to tune the density of hydrogel-like GOQD membranes to control drug release rates offers guidelines for tailoring drug release rates in hydrogel-based insulin and therapeutic delivery applications [120].

**Figure 5:**
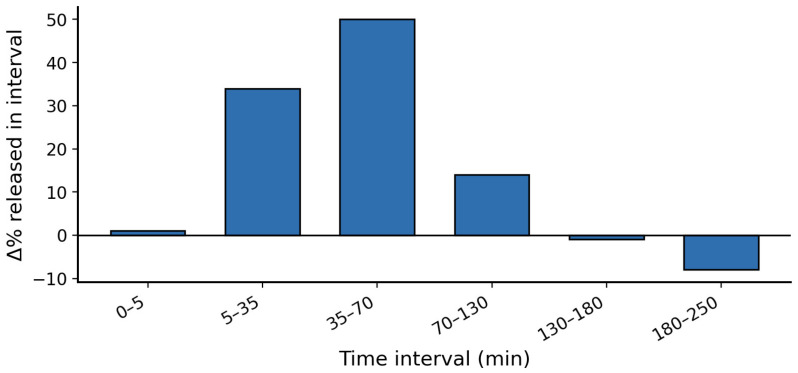
The release profile of insulin from composite carbon quantum dots (CCQDs) showing that most of the insulin is released from the scaffold about 35 to 70 min after administration. The figure was replotted using the data reported by Ref. [121].

Oral delivery of insulin was designed by Camlik et al. [121], where they reported insulin-loaded N-doped CQD combined with PEG and methylcellulose (composite-CQD abbreviated as CCQD) with a particle size of 2–10 nm, entry 2, in Table 1 [121]. Insulin release profiles of CCQDs (Figure 5) were assessed in phosphate buffer at pH 7.4 at 37 °C. A controlled release was not observed with more than 70% of insulin released within 1 h. Blood glucose levels were measured using a streptozotocin (STZ)-induced rat model for Type 1 or Type 2 diabetes. The CCQD was orally delivered without insulin, exhibiting subtle hypoglycemic effects (~20%). When combined with insulin, the oral formulation (100 IU/kg) showed significant hypoglycemic activity (~40%). This result outperformed the hypoglycemic activity (~30%) of subcutaneous insulin (50 IU/kg) over 5 h of measurement. Composite-CQD also exhibited great shelf stability for a long period of storage. After 3 months of storage at each temperature, −20, −5, 25, and 40 °C, the particle size increased from 2 nm to no higher than 11 nm. Thus, although the delivery of insulin by GQDs has not been as explored as extensively the delivery with other nanotechnology, these studies do demonstrate that GQDs can successfully be used for treatment of diabetes [132].

### 6.2. Metformin-Loaded Graphene Quantum Dots

**Figure 6:**
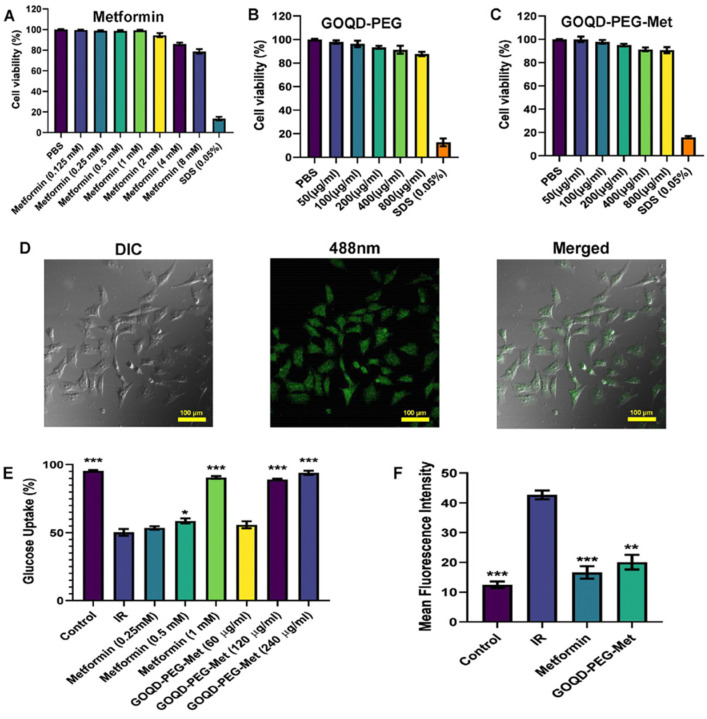
(**A**–**C**) MTT assay of PBS, metformin, GOQD-PEG and GOQD-PEG-Met in PA (palmitic acid)-induced HepG2 cells; (**D**) confocal laser scanning microscopy images are shown for metformin, GOQD-PEG and GOQD-PEG-Met uptake in HepG2 cells; (**E**) glucose uptake assay and dose selection of GOQD-PEG-Met and metformin in PA-induced in vitro insulin-resistant model in HepG2 cells; and (**F**) BODIPY staining for lipid accumulation was done in a study in PA-induced HepG2 cells upon GOQD-PEG-Met and metformin treatment. The statistics are *n* = 3, *p* values * 0.05, ** 0.01, *** 0.001, and the abbreviations are GOQD—graphene oxide quantum dots, PEG—polyethylene glycol, Met—metformin, and IR—insulin resistance. Figure 6 was published by the Public Library of Science (PLOS) in 2024 and the image is licensed under CC-BY 4.0 in Ref. [103].

Sarkar et al. [103] reported a study using an in vitro insulin resistant model platform formed by the treatment of HepG2 cells by palmitic acid (PA). In these cells, significant reduction in glucose uptake by the insulin receptor (IR) is observed compared to control cells, entry 4 in Table 1 and in Figure 6. The model system was created by the treatment of HepG2 cells by palmitic acid (PA). The HepG2 cells with reduced glucose uptake were treated with metformin, GOQD-PEG, and GOQD-PEG-metformin (GOQD-PEG-Met) for 24 h during which time a sustained release of metformin was observed. The cell viability was determined in a dose-dependent manner using a MTT assay after the addition of metformin (0.25 to 4 mM), GOQD-PEG (carrier 50 μg–800 μg) and GOQD-PEG-Met (carrier 50 μg–800 μg). These results document that similar effects of metformin are obtained upon administration of a 100–1000-fold lower concentration of GOQD-PEG-Met [103]. In Figure 6D, confocal laser scanning microscopy images are shown upon treatment with metformin, GOQD-PEG and GOQD-PEG-Met at 488 nm, documenting the uptake of GOQD-PEG-Met in the PA-induced in vitro insulin-resistant model in HepG2 cells. In Figure 6E,F, glucose levels and BODIPY staining for lipid accumulation are observed by metformin and GOQD-PEG-Met as a function of dose in PA induced in HepG2 cells. Upon metformin and GOQD-PEG-Met treatment, a decrease in either glucose or lipid levels is found and both show that treatment by free metformin is 100–1000-fold decreased compared to GOQD-PEG-Met. In summary, these results demonstrate that the metformin-loaded GOQD-PEG successfully restored both glucose and lipid uptake in insulin resistant HepG2 cells and reversed insulin resistance at a concentration 100–1000 times less than free metformin [103]. Although the specific mechanism for why the GOQD-PEG-Met is so much more effective than the free metformin is not studied extensively, we suggest that the enhancement of the efficacy of metformin is due to several factors, including that GOQD-PEG-Met is able to reach potential targets quicker than free metformin and that less metabolism of free metformin takes place when protected by the GOQD-PEG carrier.

Sarkar et al. also used a hyaluronic acid-graphene oxide quantum dots (GOQD-HA) nanocomposite for tissue-specific delivery of metformin [46], entry 5 in Table 1. Metformin-loaded hyaluronic acid-graphene oxide quantum dots (GOQD-HA-Met) successfully downregulated the expression of proinflammatory cytokines and restored antioxidant status at lower doses than free metformin in both PA-induced RAW264.7 cells and diet-induced obese mice. The GOQD-HA nanocarrier enhanced the efficacy of therapeutic metformin primarily by acting as a therapeutic agent but also from the low-grade anti-inflammatory effect of the GOQD-HA carrier [46].

Cong et al. developed metformin-loaded glucose-sensitive acrylic graphene oxide hydrogels with a particle size of 75 nm [122], entry 6 in Table 1. In vitro drug release of the metformin-loaded nanogel was assessed from 7 mM glucose concentrations to 40 mM at physiological pH over 50 h. As the concentration of glucose increased, both the swollen degree (SD) and drug release amount increased. This result was attributed to the glucose sensitivity of the nanogel, whose pore enlargement at higher glucose concentrations led to increased metformin release at a slow and controlled rate [122].

Chengnan et al. designed near-infrared (NIR) light-sensitive hydrogels which were combined with a reduced graphene oxide platform for metformin delivery [123], entry 7 in Table 1. The effect of NIR heating on metformin release was determined using GOD-metformin gels in PBS (pH 7.4) with a pore size of 25 nm. When activated by a NIR laser every 2 h, a controlled release was observed over 12 h in comparison to a non-activated passive release that had a much smaller drug release throughout the measurement. Ex vivo release profiles of rGOD-COOH/metformin gel were also studied, using mice skin and Franz diffusion cells. Without laser activation, 50 ± 5 μg cm^−2^ (10%) metformin crossed mice skin after 24 h. Laser activation with 0.7 W cm^−2^ for 10 min led to skin permeation of 56 ± 5 µg cm^−2^ (12%) metformin after 6 h, and 222 ± 10 µg cm^−2^ (45%) metformin after 24 h [123]. These studies demonstrate how an appropriately designed hydrogel can enhance drug delivery.

**Figure 7:**
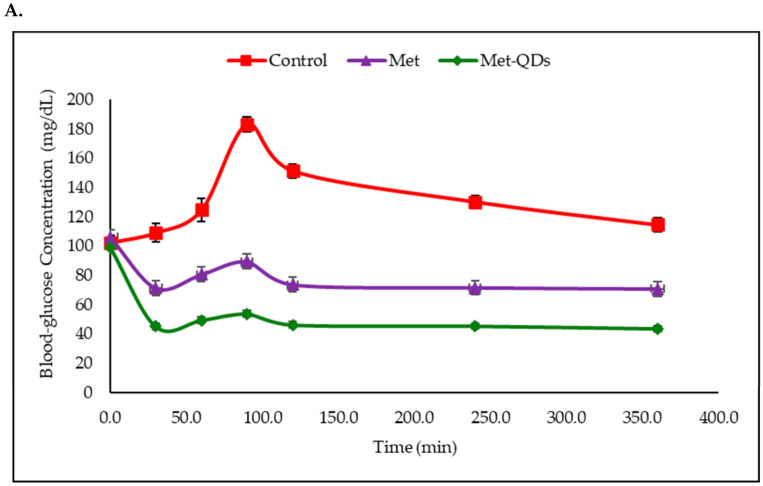

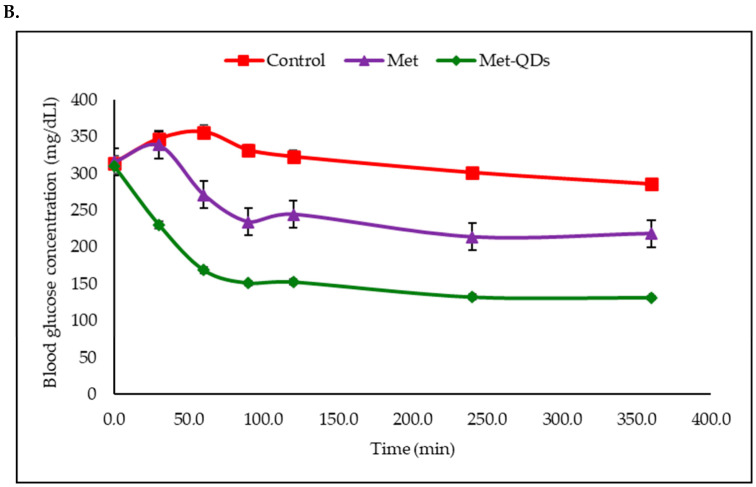
Fasting blood glucose test of normal and diabetic rats. In normal control animals the blood glucose level is shown in the red curve, animals treated with metformin are shown in the purple curve and animals treated with N-doped-CQD-Met are shown in the green curve (**A**). Fasting blood glucose test in diabetic rats showing the blood glucose level in diabetic animals in the red curve, the metformin treated animals in the purple curve and treatment with the Met on the N-doped-CQD-Met is shown in the green curve (**B**). Figure 7 was published by MDPI in 2024 in Ref. [124] and licensed by CC-BY 4.0.

Oral delivery of metformin-loaded CQD was reported by [124] and is summarized in entry 8 Table 1 and Figure 7. The CQD was prepared from citric acid and metformin resulting in metformin-loaded N-doped carbon quantum dots (N-doped CQDs) with a particle size of 9.02 nm. A fasting blood glucose test was performed both on normal Wistar albino rats and STZ-induced diabetic Wistar albino rats over 350 min. Normal rats showed blood glucose increase at about 100 min for the untreated rats followed by approaching normal levels at 350 min. Treatment with metformin and CQD-Met show blood glucose levels below normal, with the CQD-Met showing stronger hypoglycemic activity than metformin alone. Treatment of STZ-induced rats diabetic animals compared the non-treated diabetic animal with the metformin treated and the CQD-Met treated animals given 250 mg/kg orally. The CQD-Met treatment reduced the blood glucose concentration by about 50% compared with the diabetic untreated control and maintained a steady decline up to 350 min. For comparison, the free (native) metformin decreased blood glucose by about 23% given the same dosage. Histopathological tests were performed using H&E staining on the pancreatic and liver tissue of diabetic rats. With CQD-Met-treated pancreatic tissue, the stained image showed more pronounced regeneration in exocrine epithelium, islets, and connective tissue between lobules, indicating structural recovery rather than additional damage. The stained image of the liver tissue exhibited a normal trabecular network, decreased liver fat, and increased mitotic activity in hepatocytes, suggesting improved liver status compared to diabetic control.

These studies demonstrate that application of metformin that already is such a successful antidiabetic drug even further is enhancing the therapeutic properties of metformin [132,133,134].

### 6.3. Sulfonylureas-Loaded Graphene Quantum Dots

**Figure 8:**
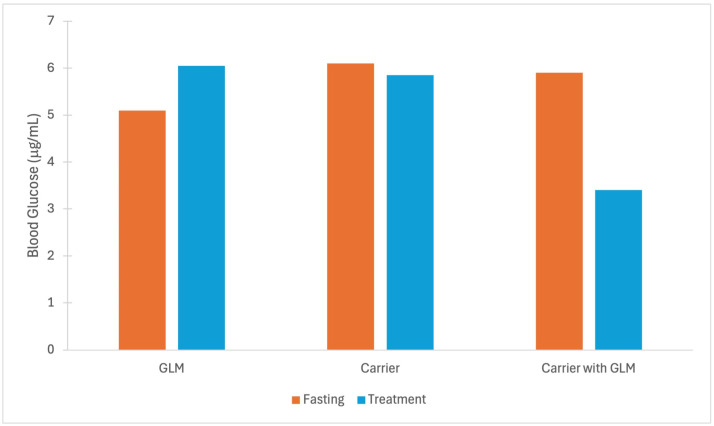
Histograms of fasting blood glucose levels before gel subcutaneous device was implanted (orange columns) and 120 h after the gel subcutaneous device with GLM, GOD carrier or GOD carrier loaded with GLM of blood glucose levels was implated (blue columns). *Y*-axis denotes blood glucose values in mmol/L. Error bars shown in original work; only the fasting blood glucose and carrier with GLM were reported with statistical significance. The plot was redrawn using the data reported by [53].

Clinical effectiveness of the oral form of glimepiride is sufficiently poor, and the development of more efficacious sulfonylureas for oral delivery to Type 2diabetes mellitus patients has continued; recently, Li et al. have investigated the improvement by suitable CD nanocarriers (entry 10) [53]. Clinical available third-generation glimepiride efficacy is improved for in situ gel subcutaneous implants by formulation of a CD in a hydroxybutyl chitosan (HBC) hydrogel, shown in Figure 8 [53]. The HBC exhibits low mechanical strength and gel instability under physiological conditions, and in combination with the rigid structure of the GQD, the hydrogel-containing glimepiride is further stabilized. The combined formulation was found to increase half-life (mean residence time) by a factor of three, extending the residence time of glimepiride in the body of the diabetic rats. Fasting blood glucose levels were measured before and after 120 h following the surgical addition of the implant and are shown in Figure 7. Fasting blood glucose levels were observed for extended periods as appropriate for in situ gel subcutaneous implants [53]. Blood glucose levels were found to be stable within a normal range. These results are also consistent with Yu et al.’s report on gelatin-coated mesoporous silica NPs (154 nm) loaded with glimepiride, which showed marked improvement to treatment by glimepiride alone because blood glucose levels decreased steadily over time [135].

### 6.4. Metformin-Loaded Fullerenes and Gadofullerene

Recent advances using fullerenes have provided new opportunities for diabetes therapy and diagnosis [5]. Several reports have been made with computational studies examining the effect of the interaction of the drug with fullerene using computational studies [136]. Reports showed effects are enhanced in the presence of metal ions [125] or when fullerenes are made out of elements other than carbon such as boron and nitrogen or aluminum and nitrogen [137], and in these studies mainly metformin has been added. Therefore, we have added a general entry (entry 7) in Table 1 citing a couple of references to such studies of hallmark diabetic parameters (blood glucose lowering, insulin levels (insulin resistance or fasting blood glucose tolerance test) or the glycated hemoglobin (HbA1c)). However, more studies were reported in which the binding affinity between the fullerenes and the drug were investigated using computational methods [5,125,136,137].

However, a remarkable study with “gadofullerene” derivatives, described in entry 11 [126], concerned the development of a functionalized C_82_ fullerene coupled with gadolinium based on gadofullerene nanocrystals that were reported earlier as a promising tumor vascular-targeting therapeutic technique [127]. Application of the functionalized gadofullerene for treatment of diabetes was done using two Type 2 diabetes mellitus animal model systems, the obese db/db mouse and the non-obese mouse model [126]. The authors found a significant improvement of impaired glucose tolerance, insulin sensitivity, and hyperglycemia after intraperitoneal administration with gadofullerene for weeks of treatment. Importantly, blood glucose levels decreased in a dose-dependent manner, and furthermore, the restored blood glucose levels persisted for ten days after the gadofullerene treatments were finished.

### 6.5. Vanadium-Containing Compounds Loaded on GQDs

Vanadium salts and one vanadium coordination complex had previously been in clinical trials but were abandoned because of toxicity issues at the concentrations needed to observe the beneficial anti-diabetic effects [55,65]. A strategy to improve hypoglycemic effects while reducing metal toxicity was reported in a series of papers using GQDs [6,7,68]. The first publication by Du et al. [68] in 2020 designed and synthesized a novel GQD-VO(*p*-dmada) complex for testing as anti-diabetic agents, entry 12 in Table 1. The in vitro tests showed that GQD-VO(*p*-dmada) transversed the cellular membrane as well as free GQDs with reduced cytotoxicity [68]. The GQD alone was found to show low but significant effects on blood lipid levels of the db/db mice [68]. Furthermore, in vivo tests were conducted on the widely utilized animal model referred to as the db/db mouse which is genetically predisposed to develop conditions like obesity and diabetes. These studies demonstrated that the GQD-VO(*p*-dmada) system exhibits a delayed glucose-lowering profile. However, more extensive studies after the three-week treatment observed greater effects on insulin enhancement and β-cell protection compared to VO(*p*-dmada) alone [68]. Overall, GQD-VO(*p*-dmada) shows improved pharmacokinetic performance and hypoglycemic effects, suggesting potential for using GQDs as a nanoplatform for drug delivery.

Vanadium compounds inhibit phosphatases and as such affect signal transduction and impact diseases such as diabetes, cancer, and substance abuse [55,61,138,139]. Since vanadium compounds are transition state analogs for phosphate esters hydrolysis, dogma predict that vanadium compounds could not be selective inhibitors for phosphatases [59]. To overcome this issue, Crans and coworkers relied on an X-ray structure reported by Hengges’ group that showed vanadate formed a transition state complex with PTP1B in the active site [6,140]. At the same time, the vanadate formed an ester with a hexapeptide that extended from the active site to an adjacent site where it was bound through several H-bonds. This vanadate ester was therefore specific to PTP1B. Using this strategy, Feng et al. [6] report the design of a complementary peptide-like ligand (PL-1 for PTP1B) that, upon coordination to vanadate (Figure 2D_1_,D_2_) forming a vanadate ester (Figure 2G), is found to be a selective inhibitor for PTP1B, entry 13 in Table 1. Alternatively, if the peptide-like ligand PL-2 coordinated to vanadate (Figure 2D_1_,D_2_) forming a vanadate ester (Figure 2H), was specific for T-cell PTPase, also entry 13 in Table 1. However, since the vanadate esters are labile in aqueous solution, the GQD was used to stabilize the vanadate ester to facilitate drug absorption. Feng et al. report formulation of the vanadate-PL1 complex on GQDs with the particle size of 2.5 nm [6]. This formulation upon administration to db/db mice produced significant improvements in fasting blood glucose levels when compared with diabetic controls. The effects observed with GQD preparation showed similar reduction in fasting glucose levels compared to the effect reported with BMOV treatment [65]. This work demonstrates that vanadate esters can be stabilized on the GQD platform and, after penetration of the cell, the vanadate ester is able to specifically find the PTB1B and carry out the inhibition and cell signaling. Thus, this work demonstrates that it is possible to design specific phosphatase inhibitors and that they work both in vitro and in vivo.

**Figure 9:**
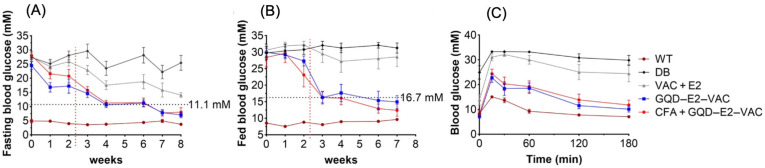
Comprehensive anti-diabetic effects of graphene quantum dots (GQD) loaded with combinations of steroid (E2), vanadyl acetylacetonate (VAC), and an antioxidant (CFA) was administered to db/db mice and different parameteris measure and compared to control and diabetic mice. (**A**) Fasting blood glucose. (**B**) Fed blood glucose levels; dashed lines indicate the levels of hyperglycemia (fasting blood glucose ≥ 11.1 mM and/or fed blood glucose ≥ 16.7 mM). (**C**) Oral glucose tolerance (OGTT) curves. Figure 9 is published by the British Pharmaceutical Society in 2024 in Ref. [7] and the images are licensed under CC-BY 4.0.

To further develop V-based compounds for treatment of Type 1 diabetes mellitus and Type 2 diabetes mellitus the therapeutic index must be increased. This can be achieved by increasing efficacy and decreasing toxicity. Shang et al. developed a GQD platform with the particle size of 2.5 nm that was able to accept both VO(acac)_2_ and 17β-oestradiol, entry 14 in Table 1 and Figure 9 [7]. Treating the db/db mice with the loaded GQD platform with both VO(acac)_2_ and 17β-oestradiol showed significant hypoglycemia effects over 8 weeks in the oral glucose tolerance test relative to the VO(acac)_2_ + 17β-oestradiol combination with and without an anti-oxidant, coniferaldehyde (CFA), shown in Figure 9. In addition, the fed glucose levels and overall glucose levels of the VO(acac)_2_ + 17β-oestradiol combination with and without CFA similarly show a large improvement compared to the diabetic and the diabetic treated with corresponding compounds without the GQD platform. This example shows that the GQD is sufficiently large to host both VO(acac)_2_ and 17β-oestradiol and that the loaded GQD will be able to enter the cells and the cargo and find the target enzyme, resulting in signaling. Importantly, this study shows that efficacy was increased and toxicity decreased, resulting in an overall increase in the therapeutic index [7].

For comparison, Liu et al. synthesized a nanocomposite containing oxovanadium(IV) and chitosan with the particle size of 70–90 nm and tested its effects on male C57BL/6J mice, 4–5 weeks old, that had been fed a high-fat high-sucrose diet, establishing an insulin-resistant mice model [141]. The V-containing nanocomposite reduced fasting blood glucose levels from the diabetic control of 11.07 mmol/L to 8.84 mmol/L, which is compared to healthy mice controls of 8.28 mmol/L. Rosiglitazone, belonging to the thiazolidinedione class of drugs, under similar treatment yielded a fasting blood glucose level of 9.17 mmol/L. In addition, blood glucose levels were measured at the end point, confirming that effects are observed for the entire study, but not determining if the chitosan was contributing to the observed anti-diabetic effect. However, Du et al. observed that the GQD alone was found to show low but significant effects on blood lipid levels of the db/db mice [6,68]. In both Shang et al.’s and Feng et al.’s publications [6,7], the presence of GQDs played a significant role, probably in protecting vanadium derivatives against digestion in the stomach and then releasing the drug into the small intestines.

## 7. Carbon NPs as Drug Carriers in Comparison with Other NPs

Early studies of GQDs-mediated drug delivery were reported in mid 2000s [142,143], so the accumulated knowledge on this topic is limited to about 20 years of studies as of 2025. Other types of NPs such as liposomes have been applied to drug delivery platforms since 1960s [144,145], leading to about 60 years of studies as of 2025. Therefore, GQDs have a less-well-established track record and, although much information have been developed based on data from many in vitro and in vivo studies [5,98], there has not been time to develop systems that could be carried into clinical trials as compared with other types of NPs such as liposomes, synthetic polymers, and biopolymers, because no clinical trial has yet been reported based on CNPs. Lacking information such as long-term toxicity effects of these systems is hindering the progress of developing CD systems towards entering clinical trials.

Preparation of all CNPs is relatively convenient, with several synthetic routes on laboratory and industrial scales, and their precursors are inexpensive as described above, making their use and future development desirable. CNPs, therefore, compare favorably with metal-based, liposomal and polymeric nanoparticles regarding access. However, there can be variability from batch to batch in the CNP product generated, so a better understanding of these processes to discover the sources of this variability still needs investigation. The fact that CDs are still not fully explored leads to unestablished protocols in diabetic drug delivery research, unlike the situation with other types of more fully understood NPs. However, as shown in this work and summarized in Table 1, the information that is available is promising and leads us to suggest that this is a growing area of research and potentially improved preparations and applications of CNPs will be discovered in the future.

Application for drug delivery of nanoparticles in general has been very successful, with many examples of drugs that become more effective and specific upon addition to a carrier; see Table 1 for specific examples and citations. However, the properties of each type of CNP vary and, as such, the ability of these systems to support a particular drug class or type varies as well, as illustrated with the images of three subtypes of CNPs in Figure 4. In general, CDs and metal-based NPs tend to carry lower drug loads compared to liposomal and polymeric NPs that encapsulate the drugs [146,147,148,149]. Similarly, drug release is less controlled and stable in CD and metal-based NPs compared to liposomal and polymeric NPs. In addition, CDs tend to favor hydrophobic drugs with high CLogP values that may lead to issues if aqueous solutions of the drugs are needed during preparation or delivery. These differences reflect the mechanisms by which the drugs are associated with the NPs because noncovalent surface association in CDs and metal-based NPs are less firm than encapsulated drugs. However, even within the group of CDs, researchers’ systems have been developed to improve drug loading [5,6,7,8,9,10]. For example, GQDs and GOQDs with their planar graphene sheets may be more able to load hydrophobic and aromatic drugs compared to the less structurally uniform CQDs prepared from bottom-up methods, as illustrated in Figure 4C. However, as mentioned above, loading can be improved by N-doping, for example, or by adding hydrogels with potential covalent attachment sites [90,91,95]. Both approaches have been used for delivery of antidiabetic agents and are described in this manuscript in Table 1. Hence, there is no doubt that CNPs have great potential and that the future will bring many more innovative methods to enhance the ability of these systems to function as drug carriers.

The potential toxicity of CNPs is an important consideration and is likely to be dependent on size and surface modification of the CNP. Wang et al. report that large-sized CNPs are more toxic than small-sized (<10 nm) CNPs [150]. Synthetic methods, either bottom-up or top-down methods, also affect the in vivo toxicity; bottom-up methods are likely less toxic, consistent with these CNPs often being smaller than those prepared from top-down methods [150]. Furthermore, bottom-up methods often employ common carbon-source precursors such as citric acid and glucose, which are derived from harmless and non-toxic materials, contributing to none or little carbon-source-based toxicity. Unsubstituted fullerenes tend to show relatively low toxicity, as anticipated from small particles, although some organ accumulation has raised concerns. Surface modification, however, can dramatically alter properties and, perhaps not surprising, there is some controversy around the toxicity of fullerenes [151]. Kuznietsova et al. report that surface modification via N-doped GQDs and trifluoromethyl-doped GQDs caused renal tubule and liver blood supply violation for multiple dosages (5 mg/kg subcutaneously) over 14 days to mice, also affecting the mortality rate and body weight [152]. Yang’s group report GQDs loaded with anti-diabetic vanadium complexes successfully reduced hepatotoxicity compared to the control without drug-GQDs loading [6,7]. Generally, CNPs derived from naturally occurring precursors such as citric acid and glucose exhibit lower toxicity than metal-based nanoparticles such as gold, silver, and cadmium [153,154], while the ultimate toxicity depends on size, surface chemistry, and dose [155].

Anti-diabetic agents have been developed since the discovery of insulin in 1921 [156]. Most patients with Type 2 diabetes mellitus prefer non-invasive oral drug administrations for their convenience [157], but alternative methods have been developed, including delivery through sublingual glands, buccal cavities, and transdermal patches. Our goal is to analyze if loading CNPs with the respective anti-diabetic drugs could improve the action of the anti-diabetic drug. Several different classes of anti-diabetic compounds that have been administered with CDs and GQDs are summarized in Table 1. Although uses of CDs and GQDs may have been more common for intravenous or intraperitoneal administration methods, we demonstrate here that successful administration of oral metformin on a CQD platform has been done (Table 1). We therefore anticipate that applications of CDs as delivery vehicles for treatment of diabetes will increase in the future, taking advantage of the desirable properties of CDs, including the redox properties [158] and photochemical activities of loaded CDs [159,160]. Theranostic applications in cancer research have been successful, enabling drug delivery, imaging, and photothermal therapy together [123,159,160], and although diabetes is a very different disease, applications in cases of urgent needs resulting from diabetic complications may be possible [114,115].

## 8. Biological Uptake of Carbon Nanocarriers With or Without Pharmaceutical Guests

**Figure 10:**
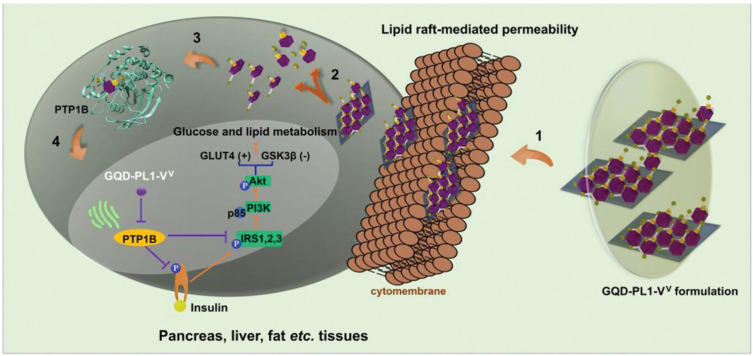
A schematic of PTP1B inhibition and cellular uptake of GQD-PL1-VV (referred to as drug-GQD) that is proposed to be absorbed through the lipid raft-mediated mechanism (step 1) by Ref. Yang et al. 2024 [7]. After entering, the drug was released into the cytoplasm in step 2 and selectively bound to PTP1B in step 3, causing inhibition of PTP1B which triggered insulin signal transduction and downstream effects in step 4. This Figure was published by Wiley and the British Pharmacological Society in 2024 in Ref. [7] and licensed under CC-BY 4.0, [69].

Cellular uptake of carbon nanoparticles, irrespective of drug loading, is tightly governed by particle size, shape, surface charge, and corona composition. Although some systems are taken up by passive diffusion endocytic pathways, most agents use ATP-requiring endocytosis. For these processes, a number of membrane binding and membrane curvature, such as clathrin, caveolae and caveolin-1, are necessary. Studies show that endocytosis is divided into two main pathways: phagocytosis and pinocytosis. Relatively large particles such as microorganisms, viruses, and macromolecules that are actin-dependent are taken up using phagocytosis [161,162]. Alternatively, pinocytosis uptakes fluids and relatively small particles that may or not be energy-dependent [161,162]. Pinocytosis can be further classified into macropinocytosis, clathrin-mediated endocytosis, caveolin-mediated endocytosis, and clathrin or caveolae-independent pathways [161,162]. Pinocytic routes have been suggested to be a primary pathway for GQDs’ energy-dependent endocytosis, while phagocytosis can be significant in macrophages [161]. However, modification of the CD by hydrogels or polymeric materials can impact how these systems enter cells. In brief, too little is known regarding these pathways, and it is likely that no single mechanism prevails for the entry of CNPs across biological membranes since so many factors can modulate the uptake process. However, to assist the readers’ conceptualization of the lipid-raft process, we show a schematic illustrating how the passive diffusion of a GQD carrier with drug load may enter cells in Figure 10 Proposed by Yang and coworkers [6,150]. The figure shows a schematic of the surface-associated drug on the GQD carrier, and after step 1, passing the membrane and being released into the cytoplasm where the drug will come off the carrier, shown in step 2. At this point, the molecule will find the target in step 3 and bind to it in step 4, initiating the signal transduction processes.

For ultrasmall CDs and fluorescent carbon nanoparticles typically in the 10–20 nm size range, multiple lines of inhibitor and temperature-dependence data indicate that pinocytic uptake is predominantly energy-dependent, involving clathrin-mediated and caveolae-mediated endocytosis [163,164]. Simultaneously, GQD and small GOQD fragments use similar pathways, where size-scaling and surface functionalization determine whether dynamin-dependent clathrin-mediated endocytosis, caveolae-mediated endocytosis, or clathrin-/caveolin (lipid rafts)-independent routes dominate [161,165]. Since the process requires ATP, if ATP is depleted, or if the temperature reaches 4 °C, the cellular uptake will terminate [166,167]. The clathrin-/caveolin-independent routes use lipid rafts. Lipid rafts are high-density regions in the membrane high on cholesterol, and pharmacologic raft disruption with cholesterol-depleting agents like methyl-β-cyclodextrin or filipin typically reduces the energy-dependent internalization of fluorescent CDs, indicating the mechanistic requirement for intact-raft architecture. Raft integrity is disrupted by cholesterol-depleting agents like methyl-β-cyclodextrin or filipin, which typically reduces the energy-dependent internalization of fluorescent CQD, indicating a mechanistic requirement for intact-raft architecture.

Drug loading on these carbon nanocarriers via adsorption, covalent conjugation, or polymer coatings modulates protein corona formation and receptor engagement, thereby biasing the balance between specific receptor-mediated pathways and non-specific pinocytosis [168]. However, typically, drug loading does not abolish the central role of dynamin-dependent clathrin and caveolae machinery in dictating internalization efficiency, intracellular trafficking (early/late endosomes and lysosomes), and ultimate cytoplasmic or nuclear access [169,170]. Further information on how these systems enter cells is paramount and such studies will help researchers understand if their drug of interest could potentially be delivered on CNPs.

## 9. Conclusions

CNPs have been developed for the past two decades since the first reports applying them for drug delivery for treatment of cancer. As a result, there is less information and reported preclinical studies on treatment of diabetes compared to treatment of cancer. CNPs, including CQDs, GQDs, GOQDs, rGOQDs, and fullerenes, are excellent candidates for drug delivery carriers, particularly those drugs that contain hydrophobic and aromatic residues. The literature reports studies of anti-diabetic drugs including insulin, metformin, glimepiride, and vanadium compounds loaded on nanoparticles and successfully used for treatment of diabetes. The use of the carbon nanoparticle scaffold includes oral and intravenous administration as well as an implant in an animal model system. The presence of the CNP generally enhances the drug’s ability to reduce elevated blood glucose levels and decrease the toxicity of the drug. Their biological properties depend on their tunable sizes, the content of sp^2^-carbon atoms, the extent of flat surfaces formed by sp^2^-carbon atoms and the surface functionalization. In this manuscript, we highlight reported studies using CNPs as carriers for anti-diabetic drugs including insulin, metformin, glimepiride, and vanadium complexes. In these studies, some anti-diabetic agents show dramatic enhancement for their therapeutic properties of the carrier-drug. This was observed with a variety of drug classes including the protein insulin, a polar small molecule metformin and a hydrophobic unstable vanadium complex. These drugs all show enhanced hyperglycemic effects at lower doses than free drugs. Although CNPs in general carry lower loads compared to liposomal or polymeric NPs, we found that modifications of the CDs by N-doping and additions of hydrogels or other additives improved CDs’ properties, overcoming these limitations. Less is understood about the mechanism by which these systems enter cells, but evidence has been presented that a graphene quantum dot carrying both hydrophobic and hydrophilic drugs as well as labile compounds was able to enter cells and reach their target. We conclude that CNPs, specifically CDs, GQD, GOQD, rGOQD, and fullerenes, are well suited to use as a drug carrier for anti-diabetic agents, because of their inherent low toxicity and the tunability of their properties. This was observed even though the shapes of these NPs vary dramatically, from flat pancakes to hollow spherical fullerenes. The limited number of studies on CNPs, with their success in a few studies, suggest that it is worthwhile applying CDs as a drug delivery system and we anticipate that their use will increase in the future in diabetes.

## Figures and Tables

**Table 1:** Studies of CNDs loaded with anti-diabetic agents.

Entry	Anti-Diabetic Agent	Type of QDs/NPs Used (Particle Size or Pore Size)	Polymer/Biopolymer/Hydrogel or Added Doping Components	Testing Method/Results	References
1.	Insulin (with human islet amyloid polypeptide (IAPP))	GOQD		Delivery of insulin and IAPP protects β-cells damage relevant to Type 2 diabetes	[119]
2.	A model peptide on GOQD was investigated to model a peptide drug release	GOQD (90 nm)	Agarose	Peptide release was monitored as a function of time	[120]
3.	Insulin	N-doped CQD (2–10 nm)	PEG, methylcellulose	Insulin release profiles, shelf-storage stability, blood glucose levels	[121]
4.	Metformin	GOQD-PEG GOQD-PEG-Met (Figure 4B) (4.27 nm)	Polyethylene glycol (PEG)	GOQD-PEG-Met treatment in vitro insulin resistance model; glucose uptake was restored and insulin resistance reversed	[103]
5.	Metformin	GOQD-HA (1.3–6.5 nm)		Restored lower antioxidant status in palmitic-acid-induced RAW264.7 cells and in diet-induced obese mice	[46]
6.	Metformin	Propylene-based GOQD (75 nm)	Hydrogel	In vivo plasma profiles, ex vivo skin permeation study, H&E staining, drug release	[122]
7.	Metformin	rGOD (25 nm)	GOD-Met hydrogel		[123]
8.	Metformin	N-doped CQD (9.02 nm)	Metformin used as doping agent	Blood glucose levels decreased 2-fold better than metformin in STZ-induced rats; brain tissue showed regeneration	[124]
9.	Glimepiride (Sulfonylurea class)	HBC-Chitosan-GO	HBC–GOQD-GLM hydrogel.	Maintaining normal blood glucose by formulation in subcutaneous implants	[53]
10.	Metformin and other drugs	Fullerenes of different types	Metformin	Both theoretical and experimental data showed interactions between drug and fullerene; some biological data showing relief of diabetic symptoms	[5,125]
11.	Gd@C82	Fullerene C82 added Gd	Gd@C82 nanocrystals were modified by H_2_O_2_ treatment to make gadofullerene	Obese db/db and non-obese mouse model with gadofullerine showed hyperglycemia, impaired glucose tolerance, and insulin sensitivity ameliorated after two or four weeks of intraperitoneal administration	[125,126]
12.	[VO(*p*-dmada)]	GQD (2.65 nm)	No	Glucose lowering of the db/db mice β-cell protection	[68]
13.	Na_3_VO_3_ (sodium metavanadate with PL1 or with PL2)	GQD (2.5 nm)	No	Specific phosphatase PTP1B inhibitor; glucose lowering of the db/db mice	[6]
14.	Vanadyl acetylacetonate (VO(acac)_2_) & 17β-oestradiol	GQD (2.5 nm)	No	VO(acac)_2_ and 17β-oestradiolson GQD decrease tocicity and increase efficacy in db/db mice	[7]

## Data Availability

Data was derived from public domain resources and Colorado State Libraries. The data presented in this study were and are available from Colorado State Library and public resources. The data bases used are Web of Science, Scifinder, Google Scholar, and Scopus.

## Abbreviations

The following abbreviations are used in this manuscript.

acac	acetylacetonate
BMOV	Bis(maltolato)oxovanadium(IV)
CDs	Carbon dots
CLogP	Calculated logarithm of the partition coefficient
CNPs	Carbon-based nanoparticles
CQDs	Carbon quantum dots
CPDs	Carbonized polymeric dots
DM	Diabetes mellitus
DPP-4	Dipeptidyl peptidase-4
GLM	Glimepiride
GLP-1	Glucagon-like peptide-1
GOQDs	Graphene oxide quantum dots
GQD	Graphene quantum dots
HA	Hyaluronic acid
HBA1c	Glycated Hemoglobin
HBC	Hydroxylbutyl
IAPP	Human islet amyloid polypeptide
Met	Metformin
N-doped	Nitrogen doped
NIR	Near-infrared
NPs	Nanoparticles
PA	Palmitic acid
PEG	Polyethylene glycol
PL1	PTP1B peptide mimic ligand
PL2	T-cell protein tyrosine phosphatase-specific ligand
PTP1B	Protein tyrosine phosphatase 1B
rGOQDs	Reduced graphene oxide quantum dots
SGLT2	Sodium–glucose cotransporter-2
STZ	Streptozotocin
VO	Vanadium oxide
VO(acac)_2_	Vanadyl acetylacetonate
